# Pneumonia

**Published:** 1894-04-07

**Authors:** 


### PNEUMONIA.

*Pathology.*—The peculiar features of acute pneumonia occurring in the aged are discussed by Dr. J. H. Pryor[1] (Buffalo). The special features are (1) its latent development, the obscurity of the symptoms, and its tendency to be very fatal. The cases occur most frequently between November and May. The upper lobe is first affected, and the disease then extends downwards. The patients rarely have the characteristic expectoration. The physical signs were apt to be very obscure, at any rate until this exudate has filled the lung to a marked extent. The countenance is anxious, the mind perturbed, and after the crisis diarrhœa often occurs. The patients sometimes die suddenly without having had severe symptoms at all.

*Aphasia*, transitory, and of an ataxic type, has been noticed by Chantimesse in a paper read before the Société Méd. des Hôp., similar to that occurring in other infectious or toxic diseases. It generally shows itself on the second or third day, the onset being either sudden and without loss of consciousness or preceded by headache and vertigo, and with partial loss of consciousness. Some cases are attended with numbness or pricking sensations on the right side of the face or in the right arm. In every case there was some paresis of the right facial nerve and right upper extremity, or even complete right hemiplegia. After a few days recovery takes place, although paresis of the right arm may persist for a time. Yet complete recovery always takes place, and therefore it is impossible to regard the phenomena as evidence of any grave organic lesion. The pathogeny of these attacks is very obscure. Two theories have been advanced—(*a*) That the symptoms are due to the direct action of the toxines on the nerve centres, (*b*) that they are due to contraction of the Sylvian artery and its branches, with resulting disturbance of the circulation in the distribution of this artery.

*Induration after lobar pneumonia* sometimes occurs. This topic forms the subject of a paper by Hekwen[2] (Chicago). Is it due to delayed resolution following pneumonia? Delafield does not believe that fibrinous pneumonia can terminate in fibroid induration; he believed that these cases are exudative and proliferative from the start, and that they are instances of a novel form of pneumonia. The author cannot accept this view. He believes that in these cases certain causes retard the normal removal of the exudate, *e.g.*, constitutional debility, local conditions, such as extensive adhesions, pleurogenous interlobular thickening, anthracosis and fibrosis of the regional lymphatic glands. While the retention of the exudate may constitute sufficient chronic tissue irritant to cause the diffuse interstitial changes, yet it is not impossible that the interstitial changes may commence very early in the attack, that the exudate may be retained on account of these changes and not *vice versa*. A case is reported by the author, illustrating the clinical and pathological character of this affection.

Dr. D. R. Paterson[3] draws attention to the effect of injury, contusions, &c., in the causation of acute pneumonia, and relates some cases in point. He emphasises the fact that the occurrence of croupous pneumonia depends largely on the favourable condition of the tissue for the growth of any infective agent.

*Treatment—Prophylactic.*—The microbal source of pneumonia is almost beyond the region of discussion now, but the application of the facts in our knowledge to the prophylaxis and treatment of pneumonia is almost fresh ground. Dr. J. Cochran[4] (Birm., Ala.) relates how an epidemic of pneumonia was checked by disinfection. The total number of cases during the epidemic was 93; total number of deaths 30, nearly one-third of the cases. The epidemic occurred in the prison at Pratt Mines, Ala. The prison was divided into three sections, and while the disinfection was going on in one section the convicts belonging to that section were crowded into the other two sections. The mattresses were taken off, and these together with the blankets were scattered over the floor. Then by means of a force-pump and a long hose-pipe the ceilings, walls, and the floors, with their contents, were literally deluged with a solution of bichloride of mercury, 1 to 1,000, until the bichloride solution stood in puddles and ran in rivulets on the floors. The mattresses, &c., were turned over so as to be wetted as thoroughly as possible on both sides. Dr. Cochran has more confidence in the disinfecting power of heat than in the bichloride. The mattresses, blankets, &c., were therefore put into large steam chambers that had been constructed for the purpose, and kept there for six hours, after which they were taken out and dried. In the meantime the disinfected wards were thoroughly scrubbed out, whitewashed, and fitted up so that they could be occupied again the next day, The convicts, before they were returned to their old quarters, were required to take a bath and to put on clean clothes. "In one week the epidemic, attacked at the period of its most rapid increase, went out like a fire under a deluge of water."

*Antitoxic Serum.*—The evidence of the correctness of the antitoxine theory, deducible from the treatment of pneumonia, is ably reviewed by Dr. J. S. Ely,[5] and G. O. F. Klemperer[6] recounts the results obtained in six cases treated by the serum of immunised rabbits, having previously tested the harmlessness of the serum injected into healthy persons. In the six cases treated the temperature fell in from six to twelve hours to the normal in four of the cases. In two cases the temperature remained normal after the initial fall, but in the others it rose again at the end of six hours or so.

Foa and Carbone[7] reported a case in which, after the first injection of 5 c.cm. of an immune rabbit's blood serum, the temperature, pulse, and respiration rate were reduced, and a second like injection on the next day brought about a crisis on the fourth day with resultant cure.

Foa soon after got together ten cases of pneumonia treated with injections of the blood serum of immune rabbits. The dose was from 5 to 7 c.cm. at a time, repeated two or three times. In eight of the cases crisis occurred within twenty-four hours after the first injection.

Foa and Scabia next used injections of dog's serum in two cases, but without good result; in fact, the disease appeared to be rather aggravated than improved. The natural immunity of the dog had been reinforced by inoculations of virulent pneumococci. Dr. Ely is of opinion that the results in these cases suggest the possibility of a difference between a natural and an acquired immunity, and that the natural immunity (*e.g.*, of a dog) is not dependent upon the constant presence in the blood of an antitoxine, as was first supposed.

Reverting to the use of rabbit serum, ten cases were reported by Janson. In five the injections were followed by fall of temperatures and other critical symptoms; in three it fell, but rose again.

Neisser tested the curative power of the serum of convalescents in other cases of pneumonia. In his first case, a young man, in the third day of a typical lobar pneumonia, in whose sputum the presence of virulent diplococci was proved, received an injection in the arm of 130 c.cm. of serum obtained by venesection from a convalescent two days after the crisis. The temperature soon sank, and in the evening became subnormal (97·7 degrees), the pulse and respiration slowed, and convalescence continued without interruption. Two other cases were treated.

Dr. Ely concludes his summary as follows: "We have thus records of thirty cases of pneumonia in which decided benefit seems to have followed the injection of serum of immune animals or of convalescents from pneumonia. If Klemperer's experiments, which resulted in the discovery of the "anti-pneumotoxin" are correct, we may fairly attribute the benefit observed to the presence of this substance in the blood injected, and we then have in the cases above referred to additional evidence of the correctness of the antitoxin theory of immunity. But it may be objected that the evidence of benefit in the cases of pneumonia is equivocal, since in many untreated cases of that disease defervescence occurs early—on the fourth, fifth, or sixth day of the disease, and that little more than this was accomplished in the test cases. The regularity with which the critical symptoms followed upon the injections after an almost uniform interval of time seems to us, however, very forcibly to suggest a relationship of cause and effect between the treatment and the amelioration of the symptoms. And it should be remembered that at best such early crisis in untreated cases of pneumonia is exceptional. At all events, the harmlessness of the treatment is demonstrated, so that we may hope soon to be possessed of enough observations to remove all doubt.

*Alcohol.*—Washbourn[8], in article on the treatment of pneumonia, in the *Journal of the American Medical Association*, enters a protest against the use of alcohol in pneumonia. Alcohol has always enjoyed the reputation of being a stimulant, but the author disbelieves this, and is firmly convinced that this view of the question will ultimately prevail. Cosgrove, reviewing the experimental work done by Ridge, Lauder Brunton, Parkes, B. W. Richardson, Prout, Fife, Vierordt, Smith, Perrin, Lehman, and others, says that, contrary to what had been and is supposed, they found that small doses of alcohol produce from the first a narcotic rather than a stimulant effect. And all these observers, with the exception of Smith, also found that alcohol in small doses diminished the amount of carbon dioxide exhaled. H. C. Wood in 1890 said that his doubts as to the stimulating effects of alcohol on the heart during anæsthesia had grown stronger and stronger for the past ten years, and that his own experiments showed that alcohol does not increase the size of the pulse or arterial pressure, but rather appears to increase the rapidity of the fall of arterial pressure, and thus hastens death. After reciting results of other investigations, the writer states, if these conclusions as to the physiological action of alcohol are warranted by the facts, we are justified in the conclusion that its use in cases of pneumonia is illogical, that it is utterly incapable of doing good, and that it is largely responsible for the high mortality rates in this disease. We ought, therefore, to abandon the use of alcoholics in the condition in question, and extend our observations in other directions in order to ascertain whether we are in possession of a therapeutic agent from whose physiological action we have any reason to expect aid in the accomplishment of the objects to be obtained, viz.: The increase of nervous sensibility and the elimination of the specific poison of the disease and the products of retrograde metamorphosis.

*Strychnine.*—"We have such an agent in strychnine, which, in its action, is diametrically opposed to that of alcohol, and one which is above all others, by reason of its physiological action, indicated in the treatment of pneumonia." Strychnine is also advocated by Dr. Simon.[9] His principle of treatment is briefly the keeping careful observations on the conditions of the heart and pulse. He gives all his cases small doses of strychnine and belladonna at the outset, and gradually increases the dose if there be any symptoms of heart failure. As a rule, he finds no other treatment necessary, beyond that which is indicated for the relief of pain and restlessness. On the question of alcohol, Dr. Simon says definitely that if for any reason the patient's power of resisting the disease is small (owing to previous disease or to an enfeebled constitution) he gives stimulants with a very free hand. "Short of the production of nausea, I am convinced that brandy may be pushed to a much larger extent than we are in the habit of doing."

*Digitalis,* given as a matter of routine, is condemned by Simon. "Digitalis has been very freely used by many practitioners throughout a case, but I do not think wisely, as there is apt to be an alarming collapse in these cases after the crisis has occurred; but I think it may very properly be given in full doses towards the end, when the heart is toiling, or any signs of œdema of the lungs is present." Dr. Reiner[10] gives a report of twenty-four cases treated by large doses of digitalis. He claims that a careful study of the tables he gives shows conclusively that digitalis is not a specific for pneumonia. The digitalis neither hindered the advance of the disease nor shortened its course, but rather protracted it There was no favourable influence on the temperature, and the pulse was not diminished in frequency. Reiner thinks that, instead of preventing collapse, it caused a slowly advancing one, which lasted a long time. Dr. Reichard[11] has found that the digitalis treatment has been somewhat unsatisfactory; he advocates carbonate of ammonia. On the other hand, the combination of pure ethyl alcohol with digitalis is, in the opinion of Dr. Huchard, the ideal treatment of pneumonia.

*Bleeding* in certain cases of pneumonia has received support from several of the authors referred to above. Thus Simon writes: "When there are signs that the right side of the heart is becoming dilated, and the ventricle is not emptying itself properly, I am every year getting more and more convinced that the proper proceeding is to bleed the patient. During the last two years I have bled in a fair number of cases with only one death occurring afterwards, and in that case my only regret is that I did not bleed earlier. The relief in every case was marked; and, without the slightest desire to return to the indiscriminate bleeding of our forefathers, I feel that we have too long neglected so ready a means of giving temporary help to a struggling heart by diminishing for a brief period the excessive amount of work."

Washbourn, admitting that bleeding in certain patients may result in good, says that the advocate of bleeding has this in his favour, that with the blood withdrawn there escapes a certain amount of the tox-albumin, and the amelioration which is claimed to follow the operation is probably due to this fact.

In a recent clinical lecture, Dr. Charles Cary[12] states that in many cases there is an extremely high arterial pressure, with hard bounding pulse, and in such cases blood-letting would do good.

*Oxygen.*—The modes of administering oxygen have recently formed the subject of a valuable contribution by Sir Benjamin Ward Richardson.[13]

[1] New York Med. Journ., Feb. 24, 1894, p. 182. [2] Chicago Clin. Rec., Jan., 1894. [3] Lancet, Jan. 20, 1894. [4] Cochran Med. Rec., Nov. 18, 1893. [5] Amer. Journ. of Med. Sci., Mar., 1894. [6] Berl. Klin. Woch., 1891, Nos. 34. 35. [7] La Rif. Med., 1891, No. 256. [8] Ther. Gaz., 1894, p. 834. [9] Birm. Med. Rev., Jan., 1894. [10] Wien. Med. Woch., 1893, Nos. 39, 40; Ther. Gaz., 1894, p. 836. [11] Med. Rec., Feb. 17, 1894. [12] Int. Clinics, 1893, vol. iv. [13] The Hospital, Jan. 6, 1894.

